# The link between tobacco smoking and susceptibility to misinformation

**DOI:** 10.1007/s00213-025-06802-1

**Published:** 2025-05-15

**Authors:** Michal Piksa, Magdalena Zaniewska, Agata Cieslik-Starkiewicz, Jonas Kunst, Mikolaj Morzy, Jan Piasecki, Rafal Rygula

**Affiliations:** 1 Maj Institute of Pharmacology Polish Academy of Sciences, Affective Cognitive Neuroscience Laboratory, 12 Smetna Street, Krakow, 31-343 Poland; 2 Department of Communication and Culture, BI Norwegian Business School, Nydalsveien 37, Oslo, N-0484 Norway; 3 Faculty of Computing and Telecommunications, Poznan University of Technology, Piotrowo 2, Poznan, 60-965 Poland; 4 Faculty of Health Sciences, Department of Bioethics, Jagiellonian University Medical College, Kopernika 40, Krakow, 31-501 Poland

**Keywords:** Tobacco, Smoking, Nicotine, Misinformation, Fake News

## Abstract

**Introduction:**

This study investigates the relationship between tobacco smoking and susceptibility to misinformation, an area that has been underexplored despite its potential implications for public health and media literacy. Smoking behavior, along with the pharmacological components present in tobacco, is often associated with habitual and cognitive patterns that may influence an individual's ability to critically evaluate and discern false information. By examining this potential link, the present study aims to shed light on the broader implications of smoking for societal challenges, such as the spread of misinformation.

**Methods:**

A quantitative *online* survey was conducted to collect data from a sample of 1,575 adult participants (M_age_ = 41.37, SD = 13.58; females: 54%, males: 46%) from the United Kingdom. Participants were categorized into three groups based on their smoking status: individuals who had smoked tobacco less than an hour before the study (n = 550), individuals who had smoked more than an hour before the study (n = 472), and non-smokers (n = 553). The survey incorporated questions assessing susceptibility to misinformation by annotating certain claims as false or true, and other instruments in order to control for impulsivity, stress level, physiological arousal and education level.

**Results:**

Smokers exhibited a lower ability to correctly recognize false claims than non-smokers. There was no difference between these groups in true news recognition.

**Discussion:**

The study, controlling for confounding factors, such as education and perceived stress, reveals that tobacco smoking may be associated with misinformation susceptibility. Further laboratory-based research should be conducted to explore the mechanisms underlying the observed relationship.

**Supplementary Information:**

The online version contains supplementary material available at 10.1007/s00213-025-06802-1.

## Introduction

In the era of information proliferation and digital communication, the ability to differentiate between accurate information and misinformation is paramount for maintaining a well-informed society. Misinformation, defined as false or misleading information shared without intent to deceive (Wardle and Derakhshan 2017), has become a growing concern, especially in the digital age where news spreads rapidly across media platforms. In contrast, fake news—which refers to deliberately fabricated or misleading information designed to resemble legitimate news content (Tandoc et al. 2018)—poses an even greater challenge to public trust and democratic stability. Both forms of false information, which broadly encompass any inaccuracies in content dissemination (Fallis 2015), have sparked concerns regarding their impact on cognitive reasoning, belief formation, and decision-making (Ognyanova et al. 2020; Ecker et al. 2024). Existing research highlights that epistemic cognition –how individuals evaluate and integrate information—plays a critical role in misinformation susceptibility. Heuristic versus analytic thinking has been extensively studied, with evidence suggesting that individuals who rely on intuitive processing rather than deliberative reasoning are more susceptible to misinformation (List et al. 2024; Pennycook and Rand 2021). However, beyond these cognitive factors, the role of psychopharmacological associations, particularly tobacco smoking and nicotine, remains underexplored (Piksa et al. 2024a).

Tobacco smoking, a widespread habit with a high risk of developing dependence, has been associated with a complex interplay of neurochemical processes that influence cognitive functions and information processing (Nadar et al. 2021; Gould 2014). Central to this interplay is nicotine, the primary psychoactive component of tobacco, known for stimulating the release of various neurotransmitters, including acetylcholine and dopamine (Barik and Wonnacott 2009; Hendrickson et al. 2013). These neurochemical changes are known to affect attention, memory, and cognitive flexibility, which are key domains in processing and evaluating the accuracy of information (Suarez et al. 2021; Oberauer 2019; Pennycook and Rand 2019). Nicotine's impact on attentional processes, for instance, might lead to altered selective attention towards information cues, potentially enhancing susceptibility to attention-grabbing but erroneous content (Mancuso et al. 1999). Moreover, the influence of nicotine on memory consolidation and retrieval (Heffernan et al. 2012) could contribute to the persistence of false information in memory, further shaping individuals'beliefs and decisions (Gould 2014). Interestingly, nicotine consumption has been linked to interpretation bias – the tendency to interpret ambiguous information in line with one’s pre-existing beliefs (Machulska et al. 2024). This phenomenon bears resemblance to confirmation bias, which plays a central role in susceptibility to misinformation (Piksa et al. 2024b). Individuals who are more prone to confirmation bias tend to selectively attend to and accept information that aligns with their worldview, potentially reinforcing distorted or polarized interpretations of reality. Despite the well-established cognitive effects of nicotine, its relationship with misinformation susceptibility has not yet been examined.

Research has shown a strong association between tobacco smoking and perceived stress—stressors increase smoking motivation at various stages, including initiation, maintenance, and relapse, while the sensation of stress relief is one of the primary reasons smokers report for their tobacco use (Bruijnzeel 2012). Physiologically, cigarette smoking has been linked with arousal levels, however, the direction of this relationship depends on specific physiological markers (Choi et al. 2015). Moreover, impulsive decision-making has been identified as a key predictor of smoking relapse (Sheffer et al. 2014), with abstinent smokers showing a higher preference for immediate rewards over delayed benefits (Mitchell 2004). Overall, these observations suggest that stress, arousal, and impulsivity play a crucial role in smoking behavior.

Understanding the relationship between tobacco smoking, nicotine, and susceptibility to misinformation is needed not only for advancing our knowledge of the cognitive processes affected by smoking but also for informing public health and communication strategies (Silver et al. 2022). By elucidating how smoking modulates cognitive functions related to misinformation processing, we can gain a better understanding of its impact on decision-making and information assessment. Developing targeted approaches to mitigate the potentially harmful effects of fake news on decision-making may be particularly important among populations more vulnerable to the negative cognitive effects of nicotine.

In this paper, we describe a quantitative *online* questionnaire study aimed at investigating the potential links between tobacco smoking and vulnerability to misinformation. To accomplish this goal, a group of adult participants from the United Kingdom was recruited through the *Prolific Academic* platform based on their smoking status. All participants underwent an online screening of their individual susceptibility to misinformation using the previously validated Misinformation Susceptibility Test – 20 (MIST-20) (Maertens et al. 2021). It is essential to emphasize that we controlled for participants'education level and accounted for additional confounders commonly linked to smoking behavior, including perceived stress, physiological arousal, and impulsivity. The present study is purely correlational and does not aim to establish a definitive causal relationship between tobacco smoking and misinformation susceptibility. Rather, it seeks to explore potential associations that could guide future experimental research.

## Material and methods

### Ethics statement

This study was conducted in accordance with the guidelines laid down in the Declaration of Helsinki, and all procedures involving research participants were approved by the Bioethics Committee of Jagiellonian University Medical College in Krakow, Poland (1072.6120.12.2022, issued on 26 January 2022). Although participants were recruited from the United Kingdom, ethical approval was obtained in Poland, where the study was designed and conducted. The study adhered to all relevant ethical guidelines for research involving international participants. Informed consent was obtained from all participants.

### Participants

A sample of 1,575 adult participants (M_age_ = 41.37, SD = 13.58) from the United Kingdom, taking no psychiatric medication, was recruited based on their tobacco usage, and further divided into 3 groups: ‘non-smokers’, and 2 groups of ‘smokers’ who were divided based on the time that elapsed from their last cigarette and beginning of the study (‘less than an hour’ and ‘more than an hour’). This division aimed to examine the effects of recent (< 1 h) versus prolonged (> 1 h) tobacco smoking on participants’ cognitive responses, particularly regarding their susceptibility to misinformation. It should be noted that the ‘< 1 h’ group could reflect both acute impact of the most recent cigarette and prolonged smoking effects, while the ‘> 1 h’ group may indicate the prolonged effects potentially influenced by temporary abstinence. Outliers (n = 36) were identified using the ROUT (Q = 1%) method and excluded from the analysis. The division described above resulted in distinction of 3 quasi-experimental groups:

1. A group that had smoked tobacco less than an hour before the study (< 1 h; n = 550);
2. A group that had smoked more than an hour before the study (> 1 h; n = 472);
3. A group of non-smokers (Control; n = 553).

### Procedure

The study was conducted between October 10th and December 6th, 2022, and was not pre-registered. Eligible participants were recruited for the study through the *online* recruitment panel platform *Prolific Academic*. Following informed consent, they were redirected to the *online* testing platform *Qualtrics.com*, where they completed the survey. The survey consisted of demographic questions (age, gender, and education level) along with question about their tobacco use patterns – *When did you smoke the last cigarette?* To get a broader image of the possible modulatory effects of tobacco smoking on susceptibility to misinformation, the participants were subjected to the following tests (listed in the order of appearance in the survey):

- The Perceived Stress Scale – 10 (PSS-10) (Cohen et al. 1983); a scale that measures perceived stress over the past month, built on a basis of ten questions with possible answers on a 5-point Likert scale, where 1 is never and 5 is very often (Cronbach’s α = 0.909. e.g., *In the last month, how often have you felt that you were unable to control the important things in your life?*).
- The Misinformation Susceptibility Test – 20 (MIST-20) (Maertens et al. 2021); a questionnaire that measures an individual’s susceptibility to misinformation that is built on a basis of 20 multithread news headlines with binominal answers true/false. The test allows one to measure individuals’ ability to recognize true (Cronbach’s α = 0.634; e.g., *One-in-Three Worldwide Lack Confidence in* NGOs) and fake news (Cronbach’s α = 0.651; e.g., *New Study: Left-Wingers Are More Likely to Lie to Get a Higher Salary*) based on a sum of correctly recognized statements as either true or false. Although discernment scores are occasionally used to assess sensitivity to misinformation, their reliance on a direct combination of two underlying measures (i.e., false and true recognition) may raise concerns about reliability and potentially distort the interpretation of results. For this reason, this approach was not applied in the present study.
- The Physiological Arousal Scale (PAQ) (Kallen 2002); a short, 6-question scale to measure individuals’ level of physiological arousal at the time of completion. The possible answers are presented on a 9-point Likert scale, where 0 stands for not at all, and 8 stands for very much (Cronbach’s α = 0.791; e.g., *In the current moment—Do you have warm or sweaty hands?*).
- The 5-Trial Adjusting Discounting Task (5-TADT) (Koffarnus and Bickel 2014); a shortened version of the Adjusting Discounting Task, where the participant chooses between receiving $500 immediately or $1000 after X times (from 1 h to 25 years). Based on the discounting threshold each participant gets his discounting value (*K*), which is interpreted as impulsivity.

### Statistical analysis

The data was analyzed using SPSS 27.0. Multivariate general linear models were computed to test for differences in fake news and true news recognition between control and smoking groups with standardization for gender, education, impulsivity, perceived stress, and physiological arousal. Post hoc analysis was made with Šidák correction. Gender, education and smoking groups were considered as independent variables, whereas impulsivity, physiological arousal and perceived stress were covariates.

## Results

Table 1 presents information on age, gender, education, raw fake news recognition score, raw true news recognition score, raw physiological arousal, raw impulsivity (K-values) and raw perceived stress score in the respective study groups.

**Table 1:** Descriptive statistics for demographic variables (age, gender, and education level), dependent variables (fake news recognition, true news recognition), and covariates (physiological arousal, impulsivity and perceived stress) presented as raw scores across the studied groups: control group, smoking less than an hour before the test (< 1 h), smoking more than an hour before the test (> 1 h) and the total sample

Group	Control	<1h	>1h	Sample
Age [mean ± SD]	45.03 ± 14.53	41.57 ± 12.53	36.86 ± 12.22	41.37 ± 13.58
Gender (male | female) [n]	236 | 317	254 | 296	238 | 234	728 | 847
Education: Less than high school degree [n]	7	14	11	32
Education: High school graduate [n]	106	132	86	324
Education: Some college but no degree [n]	84	149	105	338
Education: Associate degree in college (2-year) [n]	28	53	42	123
Education: Bachelor's degree in college (4-year) [n]	218	158	160	536
Education: Master's degree [n]	84	39	59	182
Education: Professional degree (JD, MD) [n]	8	2	7	17
Education: Doctoral degree [n]	18	3	2	23
Fake News Recognition [mean ± SD]	8.19 ± 1.89	7.59 ± 2.03	7.65 ± 1.86	7.82 ± 1.95
True News Recognition [mean ± SD]	7.62 ± 1.95	7.25 ± 2.15	7.29 ± 2.09	7.39 ± 2.07
Physiological Arousal [mean ± SD]	4.89 ± 6.21	6.23 ± 7.39	6.49 ± 7.71	5.84 ± 7.13
Impulsivity [mean ± SD]	0.21 ± 1.86	0.24 ± 1.85	0.21 ± 1.69	0.22 ± 1.81
Perceived Stress [mean ± SD]	25.36 ± 7.71	26.48 ± 7.62	26.53 ± 7.40	26.10 ± 7.60

**Fig. 1:**
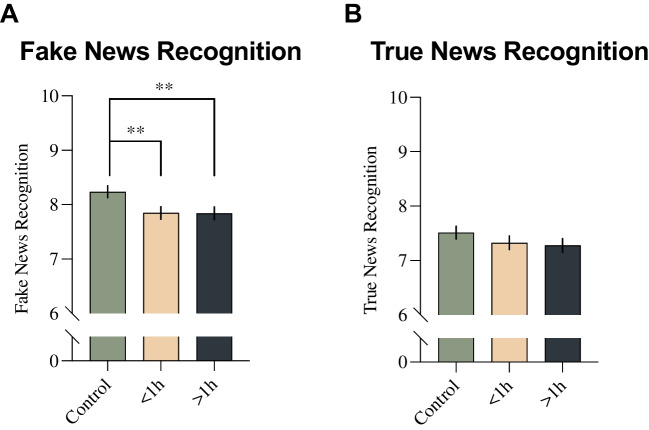
The differences between control (‘non-smokers’) and smoking groups ('< 1 h – smoked less than an hour before the test', '> 1 h – smoked more than an hour before the test') in **A** Fake News Recognition; and **B** True News Recognition. The graphs present means ± SEM estimated on standardized Gender, Education, Impulsivity, Perceived Stress and Physiological Arousal. **P < .01

A multivariate general linear model was computed for the dependent variables Fake News Recognition and True News Recognition. Among independent variables, there were Smoking Time, Gender and Education. In addition, the following covariates were included: Impulsivity, Perceived Stress and Physiological Arousal. The analysis revealed no significant Smoking Time*Education*Gender interaction (F _(70, 3052)_ = 1.181, *P* = .15) on susceptibility to either true news or fake news recognition (see Supplementary materials). Main effect of Smoking Time was significant, when controlled for each of the covariates and other variables (Pillai’s trace: F _(4, 3122)_ = 4.599, *P* = .001, η^2^ = 0.006). Between-subject tests revealed that the differences were only significant for Fake News Recognition (F _(2, 1561)_ = 7.451, *P* < 0.001, η^2^ = .009, Fig. 1A) but not for True News Recognition (F _(2, 1561)_ = 1.921, P = .147, η^2^ = 0.002, Fig. 1B). Post hoc tests with Šidák correction revealed that the control group recognized more fake news than ‘< 1 h’ (*P* = .002, D_adj_ = 0.207) and ‘> 1 h’ (*P* = .003, D_adj_ = 0.212) groups. There were no significant differences between ‘< 1 h’ and ‘> 1 h’ groups in neither Fake News Recognition (*P* = 1) nor True News Recognition (*P* = .97). More details about the statistical model can be found in the Supplementary Materials.

## Discussion

In this study, we examined the potential link between tobacco smoking and susceptibility to misinformation. Our findings indicated that, after controlling for gender, education level, impulsivity, perceived stress, and physiological arousal, smokers scored significantly lower than non-smokers in recognizing fake news. Notably, no significant difference was observed in the recognition of true news between smokers and non-smokers.

Importantly, gender, education, stress, and physiological arousal were significant predictors of misinformation susceptibility, as shown in the Supplementary Tables. However, smoking remained a significant predictor in the model even after controlling for these variables, reinforcing the robustness of the observed association between smoking and misinformation susceptibility.

We acknowledge that, in addition to the confounding factors—including gender, education level, stress, impulsivity, and physiological arousal—controlled for in our analyses, other unmeasured factors, such as memory capacity, could also contribute to the observed effects. Given these limitations, in the following discussion, we focus on a potential, hypothetical pharmacological explanation for our findings.

It is also important to emphasize that our methodology does not allow for causal inferences but rather explores the potential relationship between smoking and susceptibility to misinformation. We hope that future research will investigate this association under more controlled experimental conditions to provide further insights into the underlying mechanisms.

We hypothesized that dividing smokers into two groups—those who had smoked within the last hour (< 1 h) and those who had smoked more than an hour ago (> 1 h)—might reveal differences reflecting potential acute versus prolonged associations between tobacco use and cognitive processing. However, our results did not indicate any significant differences between these groups, suggesting that susceptibility to misinformation may be more closely associated with patterns of prolonged tobacco use rather than with its immediate effects.

In interpreting our findings, it is important to take into consideration that tobacco smoking is more prevalent among individuals with lower socioeconomic status and education levels (Nagelhout et al. 2001; Mahdaviazad et al. 2022; Hiscock et al. 2012; Garrett et al. 2019). Additionally, lower education is frequently linked to a greater vulnerability to misinformation (Hwang and Jeong 2022; Faragó et al. 2024; Chandrasekaran et al. 2024). To account for this potentially confounding factor, we meticulously adjusted performed analyses for education level, along with other potential confounders. This adjustment ensures that the observed differences between smoking and non-smoking groups are not simply due to educational disparities, demonstrating that the consequences of tobacco smoking persist independently of education level and supporting the robustness and validity of our results.

When interpreting our findings, it is tempting to hypothesize that nicotine, the primary psychoactive component in tobacco smoke, known for its reinforcing and addictive properties (Tiwari et al. 2020; Laviolette and Kooy 2004), might be responsible for the observed effects. This hypothesis aligns with existing literature suggesting that nicotine exerts complex neurocognitive effects that can impair higher-order cognitive processes, such as critical thinking and judgment (Bowirrat et al. 2012; Ramey and Regier 2019; Fried et al. 1998; Ashare and Kable 2015). One plausible explanation for our findings is the effect of nicotine on the brain's cholinergic and dopaminergic pathways. Nicotine stimulates nicotinic acetylcholine receptors, temporarily enhancing attention focus (Valentine and Sofuoglu 2018); however, chronic exposure to nicotine may lead to downregulation of these receptors, particularly those containing the β_2_ subunit, which can further result in decreased accuracy in tasks requiring attention (Picciotto and Kenny 2013). Such deficits may impair smokers' ability to carefully evaluate complex information, increasing their reliance on cognitive heuristics or ‘shortcuts’ rather than critical analysis (Kunda 1990). This reliance may lead smokers to make more biased judgements based on cues commonly associated with misinformation, such as emotional or sensational language, rather than engaging in the deeper cognitive processing required to assess the credibility of information (Martel et al. 2020; Baum and Abdel 2021; Karduni et al. 2023). The fact that we found no differences between the two smoking conditions, both of which differed significantly from the control condition, suggests that the observed effects are primarily related to prolonged tobacco use.

Indeed, nicotine's influence on the dopaminergic system may offer an explanation for smokers' heightened vulnerability to misinformation. Dopamine is central to reward processing, and chronic nicotine exposure has been shown to alter dopamine and D_2_ receptor levels (Novak et al. 2010). These alterations can lead to increased sensitivity to rewarding or emotionally charged stimuli (Biasi and Dani 2011), a trait that misinformation often exploits, particularly through sensationalized or emotionally appealing content (Kozyreva et al. 2020). As a result, smokers may be more prone to respond to such misinformation, gravitating toward information that provides immediate emotional or cognitive gratification, even if it lacks accuracy. This tendency aligns with the concept of confirmation bias (Rassin 2008), a significant factor in susceptibility to misinformation, where individuals favor information that supports their pre-existing beliefs (Piksa et al. 2024b; Bail et al. 2018). Within this framework, confirming information becomes inherently rewarding (Sharot and Sunstein 2020), reinforcing the uncritical acceptance of misinformation. Thus, the combined effects of nicotine's impact on reward pathways and confirmation bias likely contribute to the increased vulnerability of smokers to misinformation. Interestingly, a similar bias – interpretation bias, defined as the tendency to interpret ambiguous information in line with one’s pre-existing beliefs – has been shown to play a significant role in nicotine consumption (Machulska et al. 2024).

Another plausible explanation could involve smoking-induced structural and functional changes in the prefrontal cortex (PFC), a brain region, which is crucial for executive functions, including decision-making (Coutlee and Huettel 2012), impulse control (Palermo et al. 2017), and evaluative judgment (Zysset et al. 2002). Interestingly, clinical studies have demonstrated that smokers and ex-smokers exhibited hypoactivation of the PFC, including the anterior cingulate, and orbitofrontal cortices (Neuhaus et al. 2006), which could be due to the disrupted dopamine regulation of these prefrontal regions (Goldstein and Volkow 2011). Given the role of the PFC in scrutinizing and verifying information, impaired PFC function could lead to a decreased ability to discern credible information from misinformation (Moravec et al. 2022; Abe 2011). While executive dysfunction may lead smokers to act more impulsively (Fields et al. 2009), bypassing critical analysis in favor of heuristic-driven responses, it is important to note that our analysis controlled for impulsivity and physiological arousal, suggesting that the observed differences are unlikely to be caused by these factors. However, other mechanisms related to PFC function, such as emotional regulation (Suzuki and Tanaka 2021; Berboth and Morawetz 2021), may also contribute to increased susceptibility to misinformation. One could assume that the nicotine withdrawal state with its associated negative emotional and cognitive effects (Valentine and Sofuoglu 2018; Farris et al. 2015), might contribute to the reduced ability to recognize and discern fake news observed in our study. Although this hypothesis aligns with previous research linking susceptibility to misinformation with cognitive processes influenced by negative affective states (Ecker et al. 2022), the lack of difference between smokers who smoked less than an hour before the test (reflecting both acute and/or prolonged smoking effects) and those who smoked more than an hour before the test (potentially reflecting prolonged exposure combined with temporary abstinence) suggests that nicotine deprivation alone may not fully account for this effect.

Memory impairment may also play a role in smokers' decreased ability to discern the truth. Research suggests that chronic smoking disrupts prospective memory (remembering to do something, or recall details about situations in the future) (Heffernan et al. 2012, 2005), potentially leading to 'source amnesia'– a phenomenon in which individuals recall information but forget its source (Schacter et al. 1984). Subjective source reliability (Linden 2022) along with the illusory truth effect (Vellani et al. 2023) – a belief in information that has been repeated multiple times – are among the most important factors in susceptibility to misinformation. Smokers may, therefore, have a reduced ability to recall whether the information originated from a credible source, making it challenging to differentiate between reliable and unreliable information.

Last but not least, it is important to recognize that tobacco smoke contains over 4,000 compounds (Leffingwell 1999), each of which could potentially influence information processing either directly or indirectly. Indeed, contemporary studies consistently underscore the toxic effects of tobacco smoking on neurobiological functions and, consequently, on cognition (Swan and Lessov-Schlaggar 2015). Mechanisms leading to cognitive impairments due to tobacco usage may range from the direct cytotoxic effects of chemical substances released during smoking (Putnam et al. 2002), which can disrupt brain cell function, to the induction of vascular disturbances (Csiszar et al. 2009), which affect blood flow and the transport of essential nutrients to the brain. Given these factors, the specific role of nicotine in the effects observed in the current study remains not fully established and needs to be further explored.

## Limitations

Despite our best efforts, it is important to acknowledge certain inherent limitations in our study. The primary limitation pertains to our inability to establish a causal relationship between tobacco smoking and susceptibility to misinformation. While our study design enabled us to compare data gathered from individuals who self-reported tobacco smoking with those not engaged in this behavior, the specific underlying causes of the observed differences remain unknown and warrant further investigation. To address this limitation, future research should employ longitudinal experimental designs to provide a more comprehensive understanding of the interconnections between tobacco smoking, information processing, and susceptibility to misinformation.

Although our findings indicate a statistically significant relationship between smoking and susceptibility to misinformation, it is important to acknowledge the small effect size observed in our analysis (η^2^ = 0.009). While this suggests that smoking status relates to differences in fake news recognition, it also highlights that other factors, including cognitive, social, and environmental influences, likely play a more substantial role. Additionally, the observed link may have been influenced by the naturalistic nature of our study design. Future research should investigate this possibility by employing experimental designs that directly assess the impact of nicotine on misinformation susceptibility under controlled laboratory settings.

A key limitation of our study is the categorization of non-smokers, which was based on self-reported responses to the statement 'I do not smoke'. While this effectively distinguishes non-smokers from current smokers, it does not explicitly differentiate between never-smokers and former smokers. Given that former smokers may exhibit different cognitive or behavioral patterns due to previous nicotine exposure, future research should consider separating these groups to determine whether the relationships observed in this study are specific to current smokers or extend to individuals with a smoking history.

Another limitation is that other types of substance use, such as alcohol or illicit drug use, were not controlled. Given that these substances affect cognitive function, decision-making, and information processing, their potential influence on misinformation susceptibility cannot be ruled out. Future research should account for substance use history to better isolate the effects of tobacco smoking. However, this limitation does not undermine the observed association between tobacco use and increased susceptibility to misinformation.

An additional noteworthy limitation concerns the methodology employed in evaluating susceptibility to misinformation. Our study utilized a questionnaire to gauge individuals' vulnerability to (mis)information, yet it is essential to recognize that respondents' answers may not always precisely reflect their real-world behaviors, particularly in *online* social contexts. However, note that recent research shows that the scale we used in this study predicts real world behavior (Kunst et al. 2024). Nevertheless, further studies employing approaches such as machine learning to assess the behavioral support for misinformation on platforms like Twitter or Facebook are needed to verify our results.

A potential limitation of this study is that all participants were recruited from the UK, where smoking rates and regulatory environments differ from those in other countries. As of 2022, about 12.9% of UK adults were current smokers, the lowest rate since 2011, with a higher prevalence among men (14.6%) compared to women (11.2%) (Office for National S. 2023). The most significant smoking demographic is seen in the 25–34 age group, with a prevalence of 16.3%. Globally, the UK ranks as the 26th country in terms of the number of tobacco users, reflecting a moderate level of tobacco use. Additionally, the UK's strict vaping regulations contrast sharply with countries like the United States, where youth vaping is more prevalent due to less stringent advertising and product potency controls. These differences suggest that the results of our study could vary if conducted in countries with significantly different nicotine use patterns and regulatory environments. Therefore, the findings may not be directly applicable to regions with higher smoking rates or different nicotine regulation policies.

Finally, it is imperative to underscore that our current investigation employed an *online* survey method, which, although relatively novel in the domain of psychopharmacology, offers numerous advantages well-established in the realm of social sciences. These advantages encompass the ability to amass a sizable sample within a relatively brief timeframe, reduced experimental expenses, and limited ethical quandaries (Alessi and Martin 2010). Nevertheless, questions linger concerning the reliability of the smoking information provided by participants and the extent to which our findings align with those derived from traditional laboratory-based studies.

## Conclusion

This study is an early investigation into how nicotine consumption might relate to people's susceptibility to misinformation. By comparing individuals who smoke cigarettes—and thus consume nicotine—with those who do not smoke, the study found a link between smoking and an increased susceptibility to misinformation. Smokers demonstrated a somewhat reduced ability to recognize fake news. Interestingly, while there were noticeable differences in recognizing fake news and assessing their truthfulness, the ability to identify true news was the same in all tested groups. This suggests that cigarette smoking may primarily influence one's sensitivity to fake news without impacting the recognition of factual information.

The results obtained in this study are both interesting and thought-provoking. By controlling for various confounding factors, we infer that the observed differences are likely attributable to smoking itself. Our discussion highlights possible explanations, focusing primarily on the hypothesis that exposure to tobacco smoke – particularly nicotine – plays a central role in driving these effects by changing cholinergic and dopaminergic pathways. However, the causal relationship remains uncertain. While our findings indicate an association, the underlying mechanisms require further research to clarify these complex interactions.

## Supplementary Information

Below is the link to the electronic supplementary material.

ESM 1 (DOCX 47.0 KB)

## Data Availability

All data analyzed in this study have been made publicly available and can be accessed here: 10.17605/OSF.IO/59B63.
