# Supplementary material for: The link between tobacco smoking and susceptibility to misinformation
Source: Psychopharmacology (Berl). 2025 May 15;242(11):2391–400. doi: 10.1007/s00213-025-06802-1 (PMC12578774; doi:10.1007/s00213-025-06802-1)
Supplement: Supplementary file 1 — (DOCX 47.0 KB) [file 213_2025_6802_MOESM1_ESM.docx]

**3-way interaction: Smoking Time * Gender * Education**

| Effect | | Value | F | Hypothesis df | Error df | Sig. | Partial Eta Squared |
| --- | --- | --- | --- | --- | --- | --- | --- |
| Intercept | Pillai's Trace | 0.687 | 1672.058 | 2.000 | 1525.000 | 0.000 | 0.687 |
|  | Wilks' Lambda | 0.313 | 1672.058 | 2.000 | 1525.000 | 0.000 | 0.687 |
|  | Hotelling's Trace | 2.193 | 1672.058 | 2.000 | 1525.000 | 0.000 | 0.687 |
|  | Roy's Largest Root | 2.193 | 1672.058 | 2.000 | 1525.000 | 0.000 | 0.687 |
| Smoking Time | Pillai's Trace | 0.003 | 1.254 | 4.000 | 3052.000 | 0.286 | 0.002 |
|  | Wilks' Lambda | 0.997 | 1.254 | 4.000 | 3050.000 | 0.286 | 0.002 |
|  | Hotelling's Trace | 0.003 | 1.254 | 4.000 | 3048.000 | 0.286 | 0.002 |
|  | Roy's Largest Root | 0.003 | 2.418 | 2.000 | 1526.000 | 0.089 | 0.003 |
| Gender | Pillai's Trace | 0.004 | 2.999 | 2.000 | 1525.000 | 0.050 | 0.004 |
|  | Wilks' Lambda | 0.996 | 2.999 | 2.000 | 1525.000 | 0.050 | 0.004 |
|  | Hotelling's Trace | 0.004 | 2.999 | 2.000 | 1525.000 | 0.050 | 0.004 |
|  | Roy's Largest Root | 0.004 | 2.999 | 2.000 | 1525.000 | 0.050 | 0.004 |
| Education | Pillai's Trace | 0.046 | 5.088 | 14.000 | 3052.000 | <0.001 | 0.023 |
|  | Wilks' Lambda | 0.954 | 5.134 | 14.000 | 3050.000 | <0.001 | 0.023 |
|  | Hotelling's Trace | 0.048 | 5.181 | 14.000 | 3048.000 | <0.001 | 0.023 |
|  | Roy's Largest Root | 0.045 | 9.915 | 7.000 | 1526.000 | <0.001 | 0.044 |
| Impulsivity  (K-values) | Pillai's Trace | 0.002 | 1.751 | 2.000 | 1525.000 | 0.174 | 0.002 |
|  | Wilks' Lambda | 0.998 | 1.751 | 2.000 | 1525.000 | 0.174 | 0.002 |
|  | Hotelling's Trace | 0.002 | 1.751 | 2.000 | 1525.000 | 0.174 | 0.002 |
|  | Roy's Largest Root | 0.002 | 1.751 | 2.000 | 1525.000 | 0.174 | 0.002 |
| Perceived Stress  (PSS) | Pillai's Trace | 0.019 | 14.798 | 2.000 | 1525.000 | <0.001 | 0.019 |
|  | Wilks' Lambda | 0.981 | 14.798 | 2.000 | 1525.000 | <0.001 | 0.019 |
|  | Hotelling's Trace | 0.019 | 14.798 | 2.000 | 1525.000 | <0.001 | 0.019 |
|  | Roy's Largest Root | 0.019 | 14.798 | 2.000 | 1525.000 | <0.001 | 0.019 |
| Physiological Arousal (PAQ) | Pillai's Trace | 0.009 | 6.879 | 2.000 | 1525.000 | 0.001 | 0.009 |
|  | Wilks' Lambda | 0.991 | 6.879 | 2.000 | 1525.000 | 0.001 | 0.009 |
|  | Hotelling's Trace | 0.009 | 6.879 | 2.000 | 1525.000 | 0.001 | 0.009 |
|  | Roy's Largest Root | 0.009 | 6.879 | 2.000 | 1525.000 | 0.001 | 0.009 |
| Smoking Time*  Gender* Education | Pillai's Trace | 0.053 | 1.181 | 70.000 | 3052.000 | 0.146 | 0.026 |
|  | Wilks' Lambda | 0.948 | 1.180 | 70.000 | 3050.000 | 0.147 | 0.026 |
|  | Hotelling's Trace | 0.054 | 1.180 | 70.000 | 3048.000 | 0.148 | 0.026 |
|  | Roy's Largest Root | 0.029 | 1.273 | 35.000 | 1526.000 | 0.133 | 0.028 |

Design: Intercept + Smoking Time + Gender + Education + Impulsivity (K-values) + Perceived Stress (PSS) + Physiological Arousal (PAQ) + Smoking Time * Gender * Education

**2-way interaction: Smoking Time * Gender and Smoking Time * Education**

| Effect | | Value | F | Hypothesis df | Error df | Sig. | Partial Eta Squared |
| --- | --- | --- | --- | --- | --- | --- | --- |
| Intercept | Pillai's Trace | 0.680 | 1638.700 | 2.000 | 1544.000 | 0.000 | 0.680 |
|  | Wilks' Lambda | 0.320 | 1638.700 | 2.000 | 1544.000 | 0.000 | 0.680 |
|  | Hotelling's Trace | 2.123 | 1638.700 | 2.000 | 1544.000 | 0.000 | 0.680 |
|  | Roy's Largest Root | 2.123 | 1638.700 | 2.000 | 1544.000 | 0.000 | 0.680 |
| Smoking Time | Pillai's Trace | 0.002 | 0.674 | 4.000 | 3090.000 | 0.610 | 0.001 |
|  | Wilks' Lambda | 0.998 | 0.674 | 4.000 | 3088.000 | 0.610 | 0.001 |
|  | Hotelling's Trace | 0.002 | 0.673 | 4.000 | 3086.000 | 0.610 | 0.001 |
|  | Roy's Largest Root | 0.001 | 0.833 | 2.000 | 1545.000 | 0.435 | 0.001 |
| Gender | Pillai's Trace | 0.020 | 15.620 | 2.000 | 1544.000 | <0.001 | 0.020 |
|  | Wilks' Lambda | 0.980 | 15.620 | 2.000 | 1544.000 | <0.001 | 0.020 |
|  | Hotelling's Trace | 0.020 | 15.620 | 2.000 | 1544.000 | <0.001 | 0.020 |
|  | Roy's Largest Root | 0.020 | 15.620 | 2.000 | 1544.000 | <0.001 | 0.020 |
| Education | Pillai's Trace | 0.049 | 5.540 | 14.000 | 3090.000 | <0.001 | 0.024 |
|  | Wilks' Lambda | 0.951 | 5.591 | 14.000 | 3088.000 | <0.001 | 0.025 |
|  | Hotelling's Trace | 0.051 | 5.643 | 14.000 | 3086.000 | <0.001 | 0.025 |
|  | Roy's Largest Root | 0.048 | 10.636 | 7.000 | 1544.000 | <0.001 | 0.046 |
| Impulsivity  (K-values) | Pillai's Trace | 0.003 | 1.974 | 2.000 | 1544.000 | 0.139 | 0.003 |
|  | Wilks' Lambda | 0.997 | 1.974 | 2.000 | 1544.000 | 0.139 | 0.003 |
|  | Hotelling's Trace | 0.003 | 1.974 | 2.000 | 1544.000 | 0.139 | 0.003 |
|  | Roy's Largest Root | 0.003 | 1.974 | 2.000 | 1544.000 | 0.139 | 0.003 |
| Perceived Stress  (PSS) | Pillai's Trace | 0.018 | 14.096 | 2.000 | 1544.000 | <0.001 | 0.018 |
|  | Wilks' Lambda | 0.982 | 14.096 | 2.000 | 1544.000 | <0.001 | 0.018 |
|  | Hotelling's Trace | 0.018 | 14.096 | 2.000 | 1544.000 | <0.001 | 0.018 |
|  | Roy's Largest Root | 0.018 | 14.096 | 2.000 | 1544.000 | <0.001 | 0.018 |
| Physiological Arousal (PAQ) | Pillai's Trace | 0.008 | 6.489 | 2.000 | 1544.000 | 0.002 | 0.008 |
|  | Wilks' Lambda | 0.992 | 6.489 | 2.000 | 1544.000 | 0.002 | 0.008 |
|  | Hotelling's Trace | 0.008 | 6.489 | 2.000 | 1544.000 | 0.002 | 0.008 |
|  | Roy's Largest Root | 0.008 | 6.489 | 2.000 | 1544.000 | 0.002 | 0.008 |
| Smoking Time * Gender | Pillai's Trace | 0.002 | 0.776 | 4.000 | 3090.000 | 0.541 | 0.001 |
|  | Wilks' Lambda | 0.998 | 0.776 | 4.000 | 3088.000 | 0.541 | 0.001 |
|  | Hotelling's Trace | 0.002 | 0.775 | 4.000 | 3086.000 | 0.541 | 0.001 |
|  | Roy's Largest Root | 0.002 | 1.252 | 2.000 | 1545.000 | 0.286 | 0.002 |
| Smoking Time * Education | Pillai's Trace | 0.026 | 1.437 | 28.000 | 3090.000 | 0.064 | 0.013 |
|  | Wilks' Lambda | 0.974 | 1.437 | 28.000 | 3088.000 | 0.064 | 0.013 |
|  | Hotelling's Trace | 0.026 | 1.437 | 28.000 | 3086.000 | 0.064 | 0.013 |
|  | Roy's Largest Root | 0.018 | 1.980 | 14.000 | 1545.000 | 0.016 | 0.018 |

Design: Intercept + Smoking Time + Gender + Education + Impulsivity (K-values) + Perceived Stress (PSS) + Physiological Arousal (PAQ) + Smoking Time * Gender + Smoking Time * Education

**Main Effects**

| Effect | | Value | F | Hypothesis df | Error df | Sig. | Partial Eta Squared |
| --- | --- | --- | --- | --- | --- | --- | --- |
| Intercept | Pillai's Trace | 0.700 | 1816.095 | 2.000 | 1560.000 | 0.000 | 0.700 |
|  | Wilks' Lambda | 0.300 | 1816.095 | 2.000 | 1560.000 | 0.000 | 0.700 |
|  | Hotelling's Trace | 2.328 | 1816.095 | 2.000 | 1560.000 | 0.000 | 0.700 |
|  | Roy's Largest Root | 2.328 | 1816.095 | 2.000 | 1560.000 | 0.000 | 0.700 |
| Smoking Time | Pillai's Trace | 0.012 | 4.599 | 4.000 | 3122.000 | 0.001 | 0.006 |
|  | Wilks' Lambda | 0.988 | 4.610 | 4.000 | 3120.000 | 0.001 | 0.006 |
|  | Hotelling's Trace | 0.012 | 4.620 | 4.000 | 3118.000 | 0.001 | 0.006 |
|  | Roy's Largest Root | 0.012 | 9.199 | 2.000 | 1561.000 | <0.001 | 0.012 |
| Gender | Pillai's Trace | 0.019 | 15.174 | 2.000 | 1560.000 | <0.001 | 0.019 |
|  | Wilks' Lambda | 0.981 | 15.174 | 2.000 | 1560.000 | <0.001 | 0.019 |
|  | Hotelling's Trace | 0.019 | 15.174 | 2.000 | 1560.000 | <0.001 | 0.019 |
|  | Roy's Largest Root | 0.019 | 15.174 | 2.000 | 1560.000 | <0.001 | 0.019 |
| Education | Pillai's Trace | 0.053 | 6.111 | 14.000 | 3122.000 | <0.001 | 0.027 |
|  | Wilks' Lambda | 0.947 | 6.180 | 14.000 | 3120.000 | <0.001 | 0.027 |
|  | Hotelling's Trace | 0.056 | 6.248 | 14.000 | 3118.000 | <0.001 | 0.027 |
|  | Roy's Largest Root | 0.054 | 12.027 | 7.000 | 1561.000 | <0.001 | 0.051 |
| Impulsivity  (K-values) | Pillai's Trace | 0.002 | 1.749 | 2.000 | 1560.000 | 0.174 | 0.002 |
|  | Wilks' Lambda | 0.998 | 1.749 | 2.000 | 1560.000 | 0.174 | 0.002 |
|  | Hotelling's Trace | 0.002 | 1.749 | 2.000 | 1560.000 | 0.174 | 0.002 |
|  | Roy's Largest Root | 0.002 | 1.749 | 2.000 | 1560.000 | 0.174 | 0.002 |
| Perceived Stress  (PSS) | Pillai's Trace | 0.018 | 14.613 | 2.000 | 1560.000 | <0.001 | 0.018 |
|  | Wilks' Lambda | 0.982 | 14.613 | 2.000 | 1560.000 | <0.001 | 0.018 |
|  | Hotelling's Trace | 0.019 | 14.613 | 2.000 | 1560.000 | <0.001 | 0.018 |
|  | Roy's Largest Root | 0.019 | 14.613 | 2.000 | 1560.000 | <0.001 | 0.018 |
| Physiological Arousal (PAQ) | Pillai's Trace | 0.008 | 6.515 | 2.000 | 1560.000 | 0.002 | 0.008 |
|  | Wilks' Lambda | 0.992 | 6.515 | 2.000 | 1560.000 | 0.002 | 0.008 |
|  | Hotelling's Trace | 0.008 | 6.515 | 2.000 | 1560.000 | 0.002 | 0.008 |
|  | Roy's Largest Root | 0.008 | 6.515 | 2.000 | 1560.000 | 0.002 | 0.008 |

Design: Intercept + Smoking Time + Gender + Edu + Impulsivity K-values + PSS + PAQ

**(Main effects) Tests of Between-Subjects Effects**

| Source | Dependent Variable | Type III Sum of Squares | df | Mean Square | F | Sig. | Partial Eta Squared |
| --- | --- | --- | --- | --- | --- | --- | --- |
| Corrected Model | MIST_Fake | 540,107^a^ | 13 | 41,547 | 11.929 | <0.001 | 0.090 |
|  | MIST_True | 387,625^b^ | 13 | 29,817 | 7.296 | <0.001 | 0.057 |
| Intercept | MIST_Fake | 7,910,439 | 1 | 7,910,439 | 2271.274 | <0.001 | 0.593 |
|  | MIST_True | 5,809,164 | 1 | 5,809,164 | 1421.504 | <0.001 | 0.477 |
| Smoking Time | MIST_Fake | 51,901 | 2 | 25,950 | 7.451 | <0.001 | 0.009 |
|  | MIST_True | 15,701 | 2 | 7,850 | 1.921 | 0.147 | 0.002 |
| Gender | MIST_Fake | 36,702 | 1 | 36,702 | 10.538 | 0.001 | 0.007 |
|  | MIST_True | 82,970 | 1 | 82,970 | 20.303 | <0.001 | 0.013 |
| Edu | MIST_Fake | 154,575 | 7 | 22,082 | 6.340 | <0.001 | 0.028 |
|  | MIST_True | 182,003 | 7 | 26,000 | 6.362 | <0.001 | 0.028 |
| Impulsivity (K_values) | MIST_Fake | 11,691 | 1 | 11,691 | 3.357 | 0.067 | 0.002 |
|  | MIST_True | 685 | 1 | 685 | 0.168 | 0.682 | 0.000 |
| Perceived Stress  (PSS) | MIST_Fake | 96,984 | 1 | 96,984 | 27.846 | <0.001 | 0.018 |
|  | MIST_True | 6,589 | 1 | 6,589 | 1.612 | 0.204 | 0.001 |
| Physiological Arousal (PAQ) | MIST_Fake | 23,469 | 1 | 23,469 | 6.738 | 0.010 | 0.004 |
|  | MIST_True | 26,621 | 1 | 26,621 | 6.514 | 0.011 | 0.004 |
| Error | MIST_Fake | 5,436,683 | 1561 | 3,483 |  |  |  |
|  | MIST_True | 6,379,232 | 1561 | 4,087 |  |  |  |
| Total | MIST_Fake | 102,284,000 | 1575 |  |  |  |  |
|  | MIST_True | 92,792,000 | 1575 |  |  |  |  |
| Corrected Total | MIST_Fake | 5,976,790 | 1574 |  |  |  |  |
|  | MIST_True | 6,766,857 | 1574 |  |  |  |  |

a. R Squared = 0.090 (Adjusted R Squared = 0.083)

b. R Squared = 0.057 (Adjusted R Squared = 0.049)

**Smoking groups without controlling for other variables**

| Effect | | Value | F | Hypothesis df | Error df | Sig. |
| --- | --- | --- | --- | --- | --- | --- |
| Intercept | Pillai's Trace | 0.965 | 21350.800 | 2.000 | 1571.000 | 0.000 |
|  | Wilks' Lambda | 0.035 | 21350.800 | 2.000 | 1571.000 | 0.000 |
|  | Hotelling's Trace | 27.181 | 21350.800 | 2.000 | 1571.000 | 0.000 |
|  | Roy's Largest Root | 27.181 | 21350.800 | 2.000 | 1571.000 | 0.000 |
| Smoking Time | Pillai's Trace | 0.025 | 9.893 | 4.000 | 3144.000 | <0.001 |
|  | Wilks' Lambda | 0.975 | 9.949 | 4.000 | 3142.000 | <0.001 |
|  | Hotelling's Trace | 0.025 | 10.006 | 4.000 | 3140.000 | <0.001 |
|  | Roy's Largest Root | 0.025 | 20.037 | 2.000 | 1572.000 | <0.001 |

Design: Intercept + Smoking Time

**(without controlling for other variables) Tests of Between-Subjects Effects**

| Source | Dependent Variable | Type III Sum of Squares | df | Mean Square | F | Sig. |
| --- | --- | --- | --- | --- | --- | --- |
| Corrected Model | MIST_Fake | 119,087^a^ | 2 | 59,544 | 15.979 | <0.001 |
|  | MIST_True | 44,603^b^ | 2 | 22,302 | 5.215 | 0.006 |
| Intercept | MIST_Fake | 95,582,342 | 1 | 95,582,342 | 25,650.918 | <0.001 |
|  | MIST_True | 85,439,189 | 1 | 85,439,189 | 19,979.966 | <0.001 |
| SmokingTime | MIST_Fake | 119,087 | 2 | 59,544 | 15.979 | <0.001 |
|  | MIST_True | 44,603 | 2 | 22,302 | 5.215 | 0.006 |
| Error | MIST_Fake | 5,857,702 | 1572 | 3,726 |  |  |
|  | MIST_True | 6,722,254 | 1572 | 4,276 |  |  |
| Total | MIST_Fake | 102,284,000 | 1575 |  |  |  |
|  | MIST_True | 92,792,000 | 1575 |  |  |  |
| Corrected Total | MIST_Fake | 5,976,790 | 1574 |  |  |  |
|  | MIST_True | 6,766,857 | 1574 |  |  |  |

a. R Squared = 0.020 (Adjusted R Squared = 0.019)

b. R Squared = 0.007 (Adjusted R Squared = 0.005)
